# Physical activity in daily life is associated with lower adiposity values than doing weekly sports in Lc65+ cohort at baseline

**DOI:** 10.1186/1471-2458-13-1175

**Published:** 2013-12-13

**Authors:** Nadia Danon-Hersch, Brigitte Santos-Eggimann

**Affiliations:** 1 Institute of Social and Preventive Medicine (IUMSP), University of Lausanne Hospital Center, Route de la Corniche 10, Lausanne 1010, Switzerland

**Keywords:** Obesity, Adiposity, Eating habits, Daily physical activity, Stairs, Sports

## Abstract

**Background:**

Overweight and obesity prevalence is the highest at age 65–75 years in Lausanne (compared with younger classes). We aimed to describe 1) eating habits, daily physical activity (PA), and sports frequency in community-dwelling adults aged 65–70, 2) the links of these behaviors with socio-economic factors, and 3) with adiposity.

**Methods:**

Cross-sectional analysis of Lc65+ cohort at baseline, including 1260 adults from the general population of Lausanne aged 65–70 years. Eating habits (8 items from MNA) and PA (sports frequency and daily PA: walking and using stairs) were assessed by questionnaires. Body mass index (BMI), supra-iliac (SISF), triceps skin-folds (TSF), waist circumference (WC), and WHR were measured.

**Results:**

Prevalence of overweight (BMI 25.0-29.9 kg/m^2^), obesity (BMI ≥30.0 kg/m^2^), and abdominal obesity was 53%, 24%, and 45% in men; 35%, 23%, and 45% in women.

Intake of fruits or vegetables (FV) ≥ twice/day was negatively associated with male sex (prevalence 81% versus 90%, chi-square *P* < 0.001). The proportion avoiding stairs in daily life was higher among women (25%) than among men (20%, chi-square *P* = 0.003).

In multivariate analyses among both sexes, eating FV, using stairs in daily life (“stairs”), and doing sports ≥ once/week were significantly negatively associated with financial difficulties (stairs: OR = 0.54, 95% CI = 0.40-0.72) and positively with educational level (stairs: OR = 1.68, 95% CI = 1.17-2.43 for high school).

For all five log-transformed adiposity indicators in women, and for all indicators except SISF and TSF in men, a gradual decrease in adiposity was observed from category “no stairs, sports < once/week” (reference), to “no stairs, sports ≥ once/week”, to “stairs, sports < once/week”, and “stairs, sports ≥ once/week” (for example: WC in men, respectively: *ß* = −0.03, 95% CI = −0.07-0.02; *ß* = −0.06, 95% CI = −0.09- -0.03; *ß* = −0.10, 95% CI = −0.12- -0.07).

**Conclusions:**

In this population with high overweight and obesity prevalence, eating FV and PA were strongly negatively associated with financial difficulties and positively with education. Using stairs in daily life was more strongly negatively associated with adiposity than doing sports ≥ once/week.

## Background

In Switzerland, the prevalence of overweight and obesity has increased in all age groups between 1992 and 2007 [1]; in the city of Lausanne, it was the highest in age group 65–75 years in 2005, compared to younger groups, reaching a total prevalence of 73% among men, 53% among women [2]. This difference by age was also observed in the Swiss Health Survey 2012, a nationwide study using self-reported height and weight: the prevalence with body mass index (BMI) ≥25 kg/m^2^ reached 56% at age 65–74 years [3]. Overweight and obesity are important risk factors for chronic diseases and disability [4]. The youngest old deserve special attention because while the risks of obesity, chronic diseases, and disability are still present at this age, this population bears the additional risk of malnutrition, frailty [5], and sarcopenia [6,7]. In obese older adults, improving dietary habits and increasing physical activity (PA) appear to be the most effective strategies in helping to decrease body weight and improve function and survival [4,8,9]. PA has the potential to reduce the risks of both obesity [10] and sarcopenia [11]*.* To our knowledge, most randomized controlled trials focus on increasing the frequency of sports sessions, while increasing PA in everyday life in the long term is less often the target. In addition, participants are rarely asked in observational studies on PA if they usually climb stairs in their daily life.

In this context, it is important to have a more precise picture of how persons of the general population aged 65 to 70 years eat and expend energy, and how these behaviors are affected by socioeconomic factors. According to our literature review, the associations of eating habits and PA with socioeconomic position and adiposity have not been explored in detail in this age range. A positive link between socioeconomic level and PA has been described [12,13] in older adults. Concerning dietary habits, a Swiss study (CoLaus) in the same city has recently observed that the nutritional recommendations were only slightly followed in the general population (age range 40–82 years at follow-up) [14]: only 39% and 7% complied with the Swiss recommendations for fruit (≥ 2/day) and vegetables (≥ 3/day). Many studies in young adults have observed that nutrition knowledge and compliance with dietary guidelines have positive relationships with female sex, marital situation [15], high socioeconomic position [12], and higher fast food prices [16]. In Switzerland, a link between overweight and obesity and low education status has been observed in four cross-sectional National health surveys using representative samples of the Swiss population aged 18–102 years [1]; however differences in financial resources were not taken into account in this analysis. Changes in body composition are observed with advancing age [8,17], highlighting the need to monitor adiposity trends with several indicators, including BMI and waist circumference (WC) [18]. Although sophisticated methods exist for exploring adiposity, simple measures such as anthropometric indicators are useful for routine clinical practice and public health surveillance.

The present article aims to identify a hierarchy in prevention efforts: diet, versus PA; and daily PA versus sports frequency. We hypothesized that the youngest old with more or less healthy eating habits, daily PA, and sports frequency would have different anthropometric adiposity values. Our aim was to describe 1) eating habits, daily physical activity (PA), and sports frequency in community-dwelling adults aged 65 to 70, 2) the links of each of these behaviors with socioeconomic factors, and 3) with adiposity.

## Methods

### Study design and participants

The Lausanne cohort Lc65+, a study of the manifestations, determinants and outcomes of frailty [19], recruited individuals aged 65–70 in 2004, stemming from a representative sample of the general non-institutionalized population living in Lausanne (random sample from the list of all inhabitants given by the Population Office). The design of Lc65+ has already been described [19]. The ethics committee of the Faculty of Biology and Medicine of the University of Lausanne has approved the protocol. All participants provided written informed consent. Persons living in institutions or unable to respond because of advanced dementia were excluded. Of the 3,056 people who were initially mailed questionnaires, 2,096 (69%) replied, of whom 1,564 (75%) agreed to participate [19]. Overall, nonparticipants had demographic characteristics similar to those of participants [19]; only 8% of those refusing to participate attributed their refusal to poor health, and 58% had “a general reluctance to participate in any survey”. Of the 1,564 respondents to the initial questionnaire, 1,524 (97.4%) were still eligible, and 1,422 (93.3%) participated in the baseline assessment in 2005 [5,19]. This report is based on a cross-sectional analysis of the cohort at baseline (2004–5). Eating habits [20] and PA could be influenced by cognitive impairment. Therefore, 162 persons with Mini-mental State Score <24 [21] (n = 49) or missing (n = 113) were excluded and 1,260 participants remained in this analysis.

In 2004, all participants completed a questionnaire sent at home; in 2005, they underwent the assessment at the study center with an interview, measurements, and performance tests conducted by trained medical assistants.

### Eating habits and physical activity

A selection of 8 items stemming from the Mini Nutritional Assessment (MNA) [22], and describing eating habits was asked to the study population. The MNA is a widely used tool for assessing the risk of malnutrition in older adults [22].

The following MNA questions were selected (Tables 1 and 2): A) *How many full meals do you eat daily? 1) 1 meal; 2) 2 meals; and 3) 3 meals. B) Do you consume at least one serving of dairy products per day? 1) yes; 2) no. C) Do you consume at least two servings of legumes or eggs per week? 1) yes; 2) no. D) Do you consume meat, fish or poultry every day? 1) yes; 2) no. E) Do you consume two or more servings of fruits or vegetables per day? 1) yes; 2) no. F) Do you have a loss of appetite? 1) yes, severe; 2) yes, moderate; 3) not at all. G) How many drinks do you consume per day (water, juice, coffee, tea, milk, wine, beer, soup…)? 1) less than 3 glasses (1 glass = 2 dl.); 2) 3 to 5 glasses; 3) more than 5 glasses. H) Do you view yourself as being well fed, as having no nutritional problem? 1) severe malnutrition; 2) does not know or moderate malnutrition; 3) no nutritional problem.* The 8 items’ distributions are shown in Table 1.

**Table 1:** **Obesity, eating habits and physical exercise in 2004-2005**

	**Men**	**Women**	**χ**^***2***^
	**N = 519**		**N = 741**	***P*-value**
	**N (%)**	**N (%)**		
**OBESITY PREVALENCE**			
Body mass index category:			<0.001
Underweight (BMI < 18.5 kg/m^2^)	0 (0.0 )	19 (2.6 )	
Normal weight (BMI 18.5-24.9 kg/m^2^)	120 (23.3 )	290 (39.2 )	
Overweight (BMI 25.0-29.9 kg/m^2^)	271 (52.6 )	260 (35.2 )	
Obesity (BMI ≥ 30.0 kg/m^2^)	124 (24.1 )	170 (23.0 )	
Abdominal obesity (waist circumference: WC ≥ 102 cm in men, ≥ 88 cm in women)	228 (44.6 )	334 (45.3 )	0.823
WC-defined normal weight obesity (BMI < 25 kg/m^2^ and WC ≥ 102 cm in men, ≥ 88 cm in women)	0 (0.0 )	16 (2.2 )	<0.001**
WHR-defined normal weight obesity (BMI < 25 kg/m^2^ and WHR ≥ 66th gender-specific percentile)	12 (2.3 )	37 (5.0 )	0.017 **
**EATING HABITS**			
Questions from the MNA:			
Number of meals per day*			0.177
1 meal/day	33 (6.4 )	60 (8.1 )	
2 meals/day	204 (39.4 )	257 (34.8 )	
3 meals/day	281 (54.3 )	422 (57.1 )	
Meat, fish or poultry everyday	265 (51.3 )	343 (46.4 )	0.087
Dairy products ≥ once a day	425 (82.1 )	643 (86.8 )	0.021
Eggs or leguminous plants ≥ twice/week	254 (49.0 )	350 (47.2 )	0.529
“Sufficient protein intake”^†^	375 (72.5 )	514 (69.5)	0.239
Fruit or vegetables ≥ twice/day	417 (80.5 )	666 (90.1 )	<0.001
Drinks per day			0.223
<3 glasses (1 glass = 2 dl.)	40 (7.7 )	58 (7.8 )	
3 to 5 glasses	199 (38.4 )	250 (33.7 )	
>5 glasses	279 (53.9 )	433 (58.4 )	
Loss of appetite			0.627
Yes, severe	1 (0.2 )	4 (0.5 )	
Yes, moderate	25 (4.8 )	35 (4.7 )	
Not at all	492 (95.0 )	701 (94.7 )	
Self-perception of nutrition			0.708
Severe malnutrition	0 (0.0 )	0 (0.0 )	
Doesn’t know/moderate malnutrition	21 (4.1 )	27 (3.6 )	
No nutritional problem	497 (96.0 )	714 (96.4 )	
**PHYSICAL ACTIVITY (PA):**			
Daily PA:			0.003
Sitting or lying most of the time	39 (7.5 )	37 (5.1 )	
Often walking, but avoids stairs and loads	66 (12.8 )	144 (19.7 )	
Often walking and using stairs, carrying light loads	373 (72.2 )	512 (69.9 )	
Important physical activity, carries heavy loads	39 (7.5 )	40 (5.5 )	
Sports (≥20 minutes) frequency:			0.236
Less than once a week	246 (49.3 )	359 (51.9 )	
Once or twice a week	154 (30.9 )	222 (32.1 )	
Thrice a week or more	99 (19.8 )	111 (16.0 )	
**ADJUSTMENT VARIABLES:**			
Living alone (0/1)	96 (18.5 )	346 (46.7 )	<0.001
Financial difficulties (0/1)^‡^	123 (23.7 )	207 (27.9 )	0.092
Current symptoms of depression	97 (19.0 )	201 (27.7 )	<0.001
Education			<0.001
Basic compulsory	82 (15.9 )	218 (29.5 )	
Apprenticeship	225 (43.5 )	274 (37.1 )	
High school or more	210 (40.6 )	246 (33.3 )	
Current smoking^§^	119 (23.2 )	143 (19.5 )	0.112

Categorical variables are given in numbers (%); the chi-square *P*-value compares proportions across sexes.

**Fisher’s exact test.

*Instructions given to interviewers: a breakfast includes a drink and one solid food item. A lunch or dinner meal needs to include a source of proteins, starchy food (feculent), and fruit or vegetables. It can be warm or cold. A sandwich is not considered as a meal.

^†^In order to have an approximate summary measure for protein intake, “Sufficient protein intake” was defined if the participant reported eating meat, fish or poultry every day, or as an alternative, if he consumed dairy products ≥ once a day and eggs or leguminous plants ≥ twice/week.

^‡^Financial difficulties were considered if any of the following criteria was fulfilled: 1) current income clearly lower than others, 2) sometimes difficulty to make ends meet, 3) subsidy for health insurance, or 4) complementary subsidy (from old age insurance).

^§^Participants who had stopped smoking before less than one year are considered current smokers in the analyses.

**Table 2:** **Associations of eating habits with age, living arrangement, financial difficulties, symptoms of depression, and education**

	**3 meals/day (versus 1 or 2)**	**Dairy products ≥ once a day (yes versus no)**	**Eggs or leguminous plants ≥ twice/week (yes versus no)**	**Meat, fish or poultry everyday (yes versus no)**	**“Sufficient protein intake”**^**‡ **^**(yes versus no)**
	**OR [95% CI]**	**OR [95% CI]**	**OR [95% CI]**	**OR [95% CI]**	**OR [95% CI]**
**MEN**					
Age (per 1-birth year)	1.0 [0.9-1.1]	0.8 [0.7-1.0]*	1.1 [0.9-1.2]	1.1 [0.9-1.2]	1.1 [0.9-1.3]
Living alone (0/1)	0.7 [0.5-1.2]	0.7 [0.4-1.2]	0.8 [0.5-1.3]	0.9 [0.6-1.5]	0.9 [0.5-1.5]
Financial difficulties (0/1)^†^	0.6 [0.4-0.9]*	0.7 [0.4-1.1]	0.9 [0.6-1.4]	0.8 [0.5-1.2]	0.7 [0.5-1.1]
Symptoms of depression (0/1)	0.7 [0.4-1.1]	1.0 [0.5-1.8]	0.9 [0.6-1.4]	1.0 [0.6-1.6]	0.9 [0.6-1.5]
Education					
Basic compulsory	1.0 (ref)	1.0 (ref)	1.0 (ref)	1.0 (ref)	1.0 (ref)
Apprenticeship	1.0 [0.6-1.7]	2.0 [1.1-3.7]*	1.6 [1.0-2.8]	1.6 [0.9-2.7]	1.9 [1.1-3.4]*
High school or more	1.4 [0.8-2.3]	2.6 [1.4-4.9]**	1.6 [1.0-2.8]	1.2 [0.7-2.0]	1.5 [0.8-2.5]
**WOMEN**					
Age (per 1-birth year)	1.0 [0.9-1.1]	0.9 [0.8-1.1]	0.9 [0.8-1.0]	1.1 [1.0-1.2]*	1.0 [0.9-1.1]
Living alone (0/1)	0.9 [0.7-1.2]	0.8 [0.5-1.2]	0.7 [0.6-1.0]	0.5 [0.4-0.7]***	0.5 [0.4-0.8]***
Financial difficulties (0/1)^†^	0.8 [0.5-1.1]	1.1 [0.7-1.9]	0.9 [0.6-1.2]	1.0 [0.7-1.3]	0.8 [0.6-1.2]
Symptoms of depression (0/1)	0.7 [0.5-1.0]	0.8 [0.5-1.3]	1.2 [0.9-1.7]	1.3 [0.9-1.8]	1.5[1.0-2.2]
Education					
Basic compulsory	1.0 (ref)	1.0 (ref)	1.0 (ref)	1.0 (ref)	1.0 (ref)
Apprenticeship	0.9 [0.6-1.3]	1.4 [0.8-2.5]	1.2 [0.9-1.8]	1.0 [0.7-1.4]	1.1 [0.7-1.6]
High school or more	1.2 [0.8-1.8]	1.0 [0.6-1.7]	1.1 [0.7-1.6]	0.8 [0.6-1.2]	0.9 [0.6-1.3]
	**Fruit or vegetables ≥ twice/day (yes versus no)**	**> 5 glasses/day (versus ≤5 drinks/day)**	**Loss of appetite (yes versus not at all)**	**Self-perception: moderate malnutrition or doesn’t know (versus no nutritional problems)**	
	**OR [95% CI]**	**OR [95% CI]**	**OR [95% CI]**	**OR [95% CI]**	
**MEN**					
Age (per 1-birth year)	1.0 [0.9-1.2]	1.1 [0.9-1.2]	1.0 [0.8-1.4]	1.0 [0.7-1.5]	
Living alone (0/1)	0.5 [0.3-0.9]*	1.0 [0.6-1.6]	2.2 [0.9-5.4]	5.0 [1.9-13.6]**	
Financial difficulties (0/1)^†^	0.6 [0.4-1.0]*	1.0 [0.7-1.6]	1.1 [0.4-2.8]	3.2 [1.2-8.7]*	
Symptoms of depression (0/1)	0.7 [0.4-1.3]	0.9 [0.6-1.4]	2.5 [1.1-6.1]*	1.6 [0.5-4.5]	
Education					
Basic compulsory	1.0 (ref)	1.0 (ref)	1.0 (ref)	1.0 (ref)	
Apprenticeship	1.2 [0.7-2.2]	0.6 [0.4-1.0]	1.7 [0.5-6.5]	1.6 [0.4-6.4]	
High school or more	2.4 [1.2-4.5]*	0.6 [0.4-1.0]	1.0 [0.3-4.1]	0.8 [0.2-3.5]	
**WOMEN**					
Age (per 1-birth year)	1.0 [0.9-1.2]	1.2 [1.0-1.3]*	1.0 [0.8-1.3]	1.1 [0.8-1.5]	
Living alone (0/1)	0.8 [0.5-1.4]	0.8 [0.6-1.1]	1.1 [0.6-2.1]	2.5 [1.1-5.9]*	
Financial difficulties (0/1)^†^	0.5 [0.3-0.9]*	1.2 [0.8-1.7]	1.2 [0.6-2.5]	1.4 [0.6-3.3]	
Symptoms of depression (0/1)	1.2 [0.7-2.0]	1.1 [0.8-1.5]	3.4 [1.7-6.6]***	3.8 [1.7-8.5]**	
Education					
Basic compulsory	1.0 (ref)	1.0 (ref)	1.0 (ref)	1.0 (ref)	
Apprenticeship	1.2 [0.7-2.2]	1.2 [0.8-1.7]	1.0 [0.4-2.3]	1.0 [0.3-2.7]	
High school or more	1.8 [0.9-3.4]	1.0 [0.7-1.5]	1.9 [0.9-4.4]	1.6 [0.6-4.2]	

Multivariate logistic regression analysis; one separate model for each sex; approximate N = 507 for men, 724 for women.

*P < 0.05; **P < 0.01; ***P < 0.001.

^†^Financial difficulties were considered if any of the following criteria was fulfilled: 1) current income clearly lower than others, 2) sometimes difficulty to make ends meet, 3) subsidy for health insurance, or 4) complementary subsidy (from old age insurance).

^‡^“Sufficient protein intake” was defined if the participant reported eating meat, fish or poultry every day, or as an alternative, if he consumed dairy products ≥ once a day and eggs or leguminous plants ≥ twice/week.

Since the MNA does not include any quantitative assessment of total energy intake, only three questions entailing indirect (however incomplete) information about energy intake were kept for multivariate analyses of the associations between diet and PA and adiposity indicators. In order to have a simple binary variable summarizing protein intake in these analyses, “sufficient protein intake” was defined if the participant reported eating meat, fish or poultry every day, or as an alternative, if he consumed dairy products ≥ once a day and eggs or leguminous plants ≥ twice/week.

**Figure 1:**
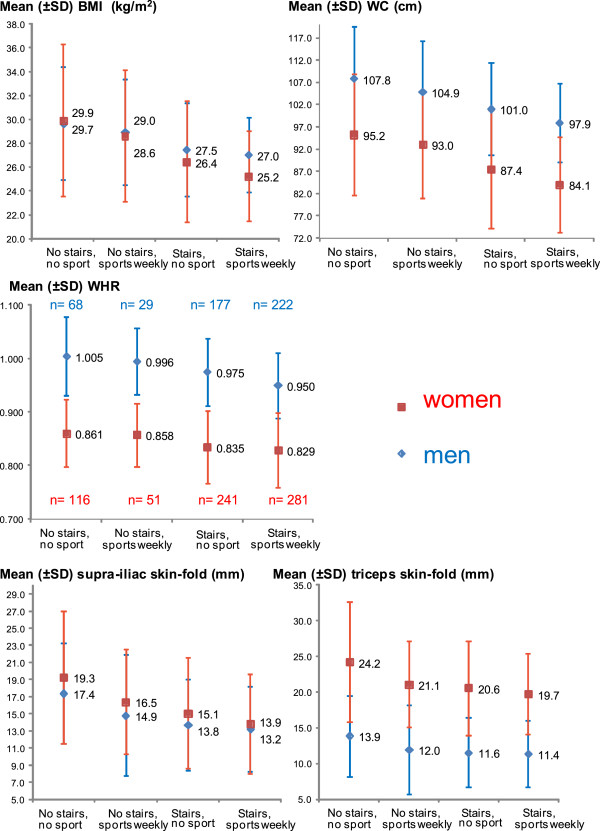
**Mean adiposity indicators according to category of physical activity (unadjusted), 2005.** Categories of variable “Daily PA and sports”: No stairs: sitting or lying most of the time, or often walking, but avoiding stairs and loads (versus often walking and using stairs, carrying light loads, and important physical activity, carries heavy loads). No sport: sports (≥20 minutes) frequency <1×/week (versus ≥1×/week).

PA was assessed with 2 main questions, described hereafter: daily PA and sports frequency (Table 3). This measurement of PA based on two questions has been adapted from the Monitoring of Trends and Determinants in Cardiovascular Disease Physical Activity Questionnaire [23,24] to suit activity patterns of individuals aged 65–70. In order to compare daily PA and sports frequency with respect to their association with adiposity, a four-category variable named “Daily PA and sports” combining both variables was created: “daily PA and sports” is a combination of a 4-category ordered variable (daily PA, further dichotomized around use, versus avoidance of stairs and loads) and a binary variable (sports frequency): A) Daily PA: “*Which statement best describes your current daily physical activity? 1) I am sitting or lying most of the time and I am not moving much; 2) I often walk, but I avoid taking stairs and carrying loads; 3) I often walk and I take stairs, I carry light loads; and 4) I make an important physical effort, I often carry heavy loads.”* In the Figure 1 and in Table 4, categories 1) and 2) of daily PA are aggregated and labeled “No stairs”; categories 3) and 4) are aggregated and labeled “Stairs”. B) Sports frequency: *“How often do you play sports for at least 20 minutes (for example, gymnastics, tennis, running, football, biking…)? <1x/week; versus ≥1/week.”*. “No sport” is the label for sports frequency <1×/week in the Figure 1 and in Table 4. “Sports weekly” is the label for sports ≥1×/week. Therefore, the 4 categories of variable “Daily PA and sports” are: I) no stairs, no sport, II) no stairs, but sports weekly, III) stairs, but no sport, and IV) stairs and sports weekly.

**Table 3:** **Association of physical activity with socio-economic and lifestyle factors**

	**Daily PA**		**Sports weekly**	
	**OR [95% CI]**	***P*-value**	**OR [95% CI]**	***P*-value**
**MEN**				
N	501		486	
Age (per 1-birth year)	1.0 [0.8-1.1]	0.623	1.0 [0.9-1.1]	0.865
Living alone (0/1)	0.5 [0.3-0.9]	0.013	0.7 [0.4-1.1]	0.094
Financial difficulties (0/1)^†^	0.4 [0.3-0.7]	0.001	0.5 [0.3-0.8]	0.002
Symptoms of depression (0/1)	0.8 [0.4-1.3]	0.367	0.9 [0.5-1.4]	0.635
Education				
Basic compulsory	1 (ref)		1 (ref)	
Apprenticeship	1.1 [0.6-2.1]	0.780	1.0 [0.6-1.7]	0.991
High school or more	1.6 [0.8-3.1]	0.150	1.4 [0.8-2.4]	0.265
Current smoking (0/1)^‡^	0.6 [0.4-1.1]	0.090	0.4 [0.3-0.7]	<0.001
**WOMEN**				
N	713		677	
Age (per 1-birth year)	1.1 [0.9-1.2]	0.373	1.1 [1.0-1.2]	0.131
Living alone (0/1)	0.8 [0.6-1.1]	0.234	1.0 [0.7-1.4]	0.978
Financial difficulties (0/1)^†^	0.6 [0.4-0.9]	0.008	0.6 [0.4-0.8]	0.002
Symptoms of depression (0/1)	0.6 [0.4-0.9]	0.017	1.0 [0.7-1.4]	0.933
Education				
Basic compulsory	1 (ref)		1 (ref)	
Apprenticeship	1.2 [0.8-1.8]	0.386	1.2 [0.8-1.7]	0.473
High school or more	1.7 [1.1-2.7]	0.018	1.7 [1.1-2.6]	0.010
Current smoking (0/1)^‡^	0.8 [0.6-1.3]	0.458	0.4 [0.3-0.7]	<0.001
**ALL**				
N	1214		1163	
Age (per 1-birth year)	1.0 [0.9-1.1]	0.675	1.0 [1.0-1.1]	0.290
Male sex	1.3 [0.5-3.2]	0.588	1.5 [0.7-3.3]	0.331
Living alone (0/1)	0.8 [0.6-1.2]	0.289	1.0 [0.7-1.4]	0.985
Financial difficulties (0/1)^†^	0.5 [0.4-0.7]	<0.001	0.5 [0.4-0.7]	<0.001
Symptoms of depression (0/1)	0.6 [0.4-0.9]	0.023	1.0 [0.7-1.4]	0.954
Education				
Basic compulsory	1 (ref)		1 (ref)	
Apprenticeship	1.2 [0.8-1.7]	0.414	1.1 [0.8-1.6]	0.443
High school or more	1.7 [1.1-2.7]	0.021	1.7 [1.1-2.6]	0.009
Current smoking (0/1)^‡^	0.8 [0.5-1.1]	0.102	0.4 [0.3-0.6]	<0.001
Living alone*male sex	0.6 [0.3-1.1]	0.115	0.7 [0.4-1.2]	0.151
Depressive symptoms*male sex	1.1 [0.6-2.2]	0.687	0.9 [0.5-1.6]	0.691
Education*male sex	1.0 [0.7-1.5]	0.951	0.9 [0.6-1.3]	0.528

*Daily physical activity (PA, 0/1):*1) Often walking and using stairs, carrying light loads, and important physical activity, carries heavy loads; 0) sitting or lying most of the time, or often walking, but avoiding stairs and loads. *Sports weekly (0/1):* Sports frequency <1×/week (0), versus ≥1×/week (1). Multivariate logistic regression analysis.

^†^Financial difficulties were considered if any of the following criteria was fulfilled: 1) current income clearly lower than others, 2) sometimes difficulty to make ends meet, 3) subsidy for health insurance, or 4) complementary subsidy (from old age insurance).

^‡^Participants who had stopped smoking before less than one year are considered current smokers in the analyses.

*Interaction.

**Table 4:** **Associations of five log-transformed adiposity indicators with eating habits and physical activity**

	**Ln (BMI)**		**Ln (WC)**		**Ln (WHR)**		**Ln (SISF)**		**Ln (TSF)**	
	***ß *** **[95% CI]**	***P***	***ß *** **[95% CI]**	***P***	***ß *** **[95% CI]**	***P***	***ß *** **[95% CI]**	***P***	***ß *** **[95% CI]**	***P***
**MEN: (N ≥ 477):**										
Eating habits:										
Three meals/day	−0.02 [−0.04-0.01]		−0.01 [−0.02-0.01]		−0.01 [−0.02-0.00]		0.00 [−0.07-0.08]		−0.04 [−0.11-0.03]	
Fruit and veg. ≥twice/day	0.00 [−0.03-0.03]		0.00 [−0.02-0.03]		0.00 [−0.02-0.01]		0.05 [−0.05-0.14]		0.02 [−0.07-0.12]	
Sufficient protein intake^$^	0.03 [0.00-0.06]	*	0.03 [0.01-0.05]	**	0.02 [0.01-0.03]	**	0.04 [−0.04-0.12]		−0.03 [−0.11-0.04]	
Daily PA and sports										
1: No stairs, no sport	0 (ref.)		0 (ref.)		0 (ref.)		0 (ref.)		0 (ref.)	
2: No stairs, sports ≥1×/wk	−0.03 [−0.08-0.03]		−0.03 [−0.07-0.02]		0.00 [−0.03-0.03]		−0.25 [−0.43- -0.07]	**	−0.22 [−0.40- -0.04]	*
3: Stairs, no sport	−0.07 [−0.11- -0.04]	***	−0.06 [−0.09- -0.03]	***	−0.02 [−0.04- -0.01]	*	−0.24 [−0.35- -0.13]	***	−0.18 [−0.29- -0.07]	**
4: Stairs, sports ≥1×/wk	−0.09 [−0.13- -0.05]	***	−0.10 [−0.12- -0.07]	***	−0.05 [−0.07- -0.03]	***	−0.30 [−0.41- -0.18]	***	−0.20 [−0.31- -0.09]	**
Age (per 1-birth year)	0.01 [0.00-0.01]		0.00 [0.00-0.01]		0.00 [0.00-0.01]		0.02 [0.00-0.05]		0.00 [−0.02-0.03]	
Living alone (0/1)	−0.03 [−0.06-0.00]		−0.01 [−0.04-0.01]		0.01 [−0.01-0.02]		0.03 [−0.07-0.12]		0.02 [−0.08-0.11]	
Financial diff. (0/1)^€^	−0.01 [−0.03-0.02]		0.00 [−0.02-0.02]		0.00 [−0.01-0.01]		−0.01 [−0.10-0.08]		0.01 [−0.07-0.10]	
Symptoms of depression	0.03 [0.00-0.06]	*	0.02 [−0.01-0.04]		0.00 [−0.01-0.02]		0.06 [−0.04-0.15]		0.04 [−0.06-0.13]	
Education										
Basic compulsory	0 (ref.)		0 (ref.)		0 (ref.)		0 (ref.)		0 (ref.)	
Apprenticeship	−0.03 [−0.07-0.00]		−0.02 [−0.05-0.01]		−0.01 [−0.03-0.01]		−0.01 [−0.11-0.10]		−0.03 [−0.13-0.07]	
≥High school	−0.05 [−0.09- -0.02]	**	−0.03 [−0.06- -0.01]	*	−0.02 [−0.04- -0.01]	*	−0.02 [−0.13-0.08]		−0.03 [−0.13-0.08]	
Current smoking (0/1)§	−0.04 [−0.07- -0.01]	*	−0.01 [−0.03-0.01]		0.01 [−0.01-0.02]		−0.11 [−0.20- -0.03]	*	−0.05 [−0.14-0.03]	
**WOMEN (N ≥ 657)**										
Eating habits:										
Three meals/day	0.00 [−0.03-0.03]		0.01 [−0.02-0.03]		0.00 [−0.01-0.01]		0.06 [−0.02-0.13]		−0.03 [−0.09-0.03]	
Fruit and veg. ≥twice/day	0.05 [0.00-0.10]	*	0.02 [−0.02-0.05]		−0.01 [−0.03-0.01]		0.14 [0.02-0.27]	*	0.14 [0.04-0.23]	**
Sufficient protein intake^$^	0.03 [0.00-0.06]		0.02 [0.00-0.04]		0.01 [−0.01-0.02]		0.04 [−0.04-0.11]		0.05 [−0.01-0.11]	
Daily PA and sports										
1: No stairs, no sport	0 (ref.)		0 (ref.)		0 (ref.)		0 (ref.)		0 (ref.)	
2: No stairs, sports ≥1×/wk	−0.04 [−0.10-0.01]		−0.02 [−0.07-0.02]		0.00 [−0.03-0.03]		−0.16 [−0.32-0.00]	*	−0.13 [−0.25- -0.01]	*
3: Stairs, no sport	−0.12 [−0.16- -0.08]	***	−0.08 [−0.11- -0.05]	***	−0.03 [−0.04- -0.01]	**	−0.25 [−0.36- -0.14]	***	−0.15 [−0.23- -0.07]	***
4: Stairs, sports ≥1×/wk	−0.16 [−0.20- -0.12]	***	−0.12 [−0.15- -0.09]	***	−0.03 [−0.05- -0.01]	**	−0.34 [−0.45- -0.23]	***	−0.19 [−0.27- -0.11]	***
Age (per 1-birth year)	0.00 [−0.01-0.01]		0.00 [−0.01-0.01]		0.00 [0.00-0.00]		0.01[−0.02-0.03]		0.00 [−0.02-0.02]	
Living alone (0/1)	−0.03 [−0.06-0.00]	*	−0.02 [−0.04-0.00]		−0.01 [−0.02-0.01]		−0.05 [−0.12-0.03]		−0.06 [−0.11-0.00]	*
Financial diff. (0/1)^€^	0.04 [0.01-0.07]	*	0.02 [−0.01-0.04]		0.00 [−0.01-0.02]		0.08 [−0.01-0.16]		0.05 [−0.01-0.12]	
Symptoms of depression	0.01[−0.02-0.04]		0.01[−0.01-0.04]		0.01[0.00-0.02]		0.06[−0.02-0.14]		0.01[−0.05-0.07]	
Education										
Basic compulsory	0 (ref.)		0 (ref.)		0 (ref.)		0 (ref.)		0 (ref.)	
Apprenticeship	−0.02[−0.05-0.01]		−0.01[−0.03-0.02]		−0.01[−0.02-0.01]		−0.04[−0.13-0.05]		−0.02[−0.09-0.05]	
≥High school	−0.06[−0.10- -0.03]	***	−0.04[−0.06- -0.01]	**	−0.02[−0.03-0.00]	*	−0.14[−0.23- -0.05]	**	−0.07[−0.14-0.00]	
Current smoking (0/1)^§^	−0.07[−0.11- -0.04]	***	−0.04[−0.06- -0.01]	**	0.00[−0.01-0.02]		−0.21[−0.30- -0.12]	***	−0.15[−0.22- -0.08]	***

Multivariate linear regression analysis. The table shows all covariates included in the adjustment.

^$^“Sufficient protein intake” was defined if the participant reported eating meat, fish or poultry every day, or as an alternative, if he consumed dairy products ≥ once a day and eggs or leguminous plants ≥ twice/week.

^€^Financial difficulties (diff.) are considered if any of the following criteria is fulfilled: 1) current income clearly lower than others, 2) sometimes difficulty to make ends meet, 3) subsidy for health insurance, or 4) complementary subsidy (from old age insurance).

^§^Participants who had stopped smoking before less than one year are considered current smokers in the analyses.

*P < 0.05; **P < 0.01; ***P < 0.001.

### Adiposity indicators

Height, weight, supra-iliac skin-fold (SISF), triceps skin-fold (TSF), waist (WC), and hip circumferences were measured without shoes. Weight was assessed with a digital SECA scale. The standard procedure recommended in NHANES III [25] was followed. WC was measured at the level midway between the lowest rib and the highest point of the iliac crest. Hip circumference was recorded as the maximum circumference over the buttocks. Skin-folds were measured with a GPM® caliper on the dominant side. Overweight and obesity were defined according to body mass index (BMI = 25.0-29.9 kg/m^2^ and ≥30.0 kg/m^2^). Abdominal obesity was considered if WC was ≥102 cm for men and ≥88 cm for women [26].

### Other Covariates

Potential confounders of the diet-adiposity relationship or the PA-adiposity association are shown in Table 1 and adjusted for in multivariate models (Tables 3 and 4): age, living arrangement (living alone, 0/1), financial difficulties, symptoms of depression, education, and smoking status are known to be associated with eating habits [15,27,28], PA (as independent variables) [12,29], and adiposity (as the dependent variable) [8]. The educational level entitled “high school or more” includes high school, professional school, or university. Living alone was assessed by the question: *“With how many persons are you currently living? 1) I am living alone; 0) I am living with … persons”.* Financial difficulties was a variable of interest for objective 2 (the cross-sectional association between eating habits, PA, and socio-economic factors), and a potential confounder for objective 3. It is defined in the footnotes of Tables 1, 2, 3, and 4. Current symptoms of depression were considered for the participants who had answered “yes” to at least one of two screening questions [30]: “*During the past month have you often been bothered by feeling down, depressed, or hopeless?”* and “*During the past month have you often been bothered by little interest or pleasure in doing things?”* Since eating habits, PA, and adiposity indicators differ between men and women [31,32], all analyses are stratified by sex.

### Statistical analyses

Results were expressed as absolute numbers and percentages. Bivariate comparisons were performed using the chi-square test or Fisher exact test for categorical variables. Table 2 shows multivariate logistic regression analyses of the cross-sectional associations between eating habits and age, living arrangement, financial difficulties, symptoms of depression, and education. Table 3 shows multivariate logistic regression analyses of the cross-sectional associations of “daily PA” and “sports weekly” with age, living arrangement, financial difficulties, depressive symptoms, education, and current smoking.

Table 4 shows multivariate linear regressions of the cross-sectional associations of each of five anthropometric adiposity indicators with eating habits and PA. Eating habits and PA are simultaneously included in the same model. Since residuals had skewed distributions, all adiposity indicators were log-transformed. All potential confounders included in the adjustment are shown in Table 4. Statistical analyses were performed using Stata 12 software (Stata Corp, College Station, TX).

## Results

### Eating habits and PA

The prevalence of underweight (BMI < 18.5 kg/m^2^) and normal weight was 0% and 23% among men, respectively 3% and 39% among women. Overweight and obesity prevalence was 53% and 24% among men, 35% and 23% among women. Abdominal obesity prevalence was 45% (both sexes). The prevalence of normal weight central obesity (BMI <25.0 kg/m^2^ and WC ≥ 102 cm for men, ≥ 88 cm for women) was 0.0% among men, and 2.2% (16/736) among women. The prevalence with BMI < 25.0 kg/m^2^ and WHR ≥ 66th gender-specific percentile was 2.3% in men and 5.0% in women (Table 1).

According to Table 1, 81% of men and 90% of women ate fruit or vegetables (FV) ≥2×/day (chi-square *P* < 0.001). 5% of both men and women reported moderate appetite loss and 4% moderate malnutrition (or unawareness of their own nutritional state).

Concerning physical activity (PA) in daily life, a higher proportion of women (25%) avoided using stairs or carrying loads than men (20%, chi-square *P* = 0.003). About half of men and women played sports <1×/week, without any significant sex difference. Women more frequently lived alone (47% vs. 19%, chi-square *P* < 0.001) and more often experienced symptoms of depression than men (28% vs. 19%, chi-square *P* < 0.001). Financial difficulties (about one quarter) and smoking (about one fifth) were equally distributed among sexes.

### Links of eating habits and physical activity with socio-economic factors

According to Table 2, eating FV ≥ twice/day was negatively associated with financial difficulties and positively with education. Among men, eating 3 meals per day was significantly negatively associated with financial difficulties (OR = 0.6, 95% CI = 0.4–0.9). Among women, eating meat, fish, or poultry every day was positively related with being younger (OR = 1.1, 95% CI = 1.0–1.2 for each additional birth year); but very strongly negatively with living alone (OR = 0.5, 95% CI = 0.4–0.7). Men living alone were less likely to eat FV ≥2×/day (OR = 0.5, 95% CI = 0.3–0.9). Self-perception of malnutrition was strongly associated with living alone in both sexes. In men, this perception was also associated with financial difficulties (OR = 3.2, 95% CI = 1.2-8.7). Symptoms of depression were strongly associated with appetite loss in both sexes and with perception of moderate malnutrition in women (OR = 3.8, 95% CI = 1.7-8.5).

Table 3 shows that both daily PA and sports weekly have strong and significant negative links with financial difficulties in both sexes, and significant positive links with educational level in women only; however the interaction was not statistically significant. Unlike women, men living alone were significantly less likely to use stairs and carry light loads in their daily life than men living with someone (OR = 0.5, 95% CI = 0.3-0.9). No sex interaction reached significance. Sports weekly, but not daily PA, had strong significant negative links with current smoking in both sexes.

Across the 4 categories of variable “Daily PA and sports”, there was a gradual decrease in the prevalence of participants living alone, experiencing financial difficulties, depressive symptoms, current smoking, and a gradual increase in educational level. The test for trend *P*-value was statistically significant (*P* < 0.05) for all above mentioned associations (Additional file 1).

### Links of eating habits and physical activity with adiposity

The Figure 1 shows univariate associations between each adiposity indicator and variable “Daily PA and sports”. Men had higher BMI, WC, and waist-to-hip ratio (WHR) mean values than women. In contrary, women had higher SISF and TSF mean values than men. For all five adiposity indicators and in both sexes, a progressive decrease in adiposity was observed from category “No stairs, no sport”, to “No stairs, sports weekly”, to “Stairs, no sport”, and finally “Stairs, sports weekly”. This consistent univariate association was statistically significant for all indicators (test for trend *P*-value <0.001 for all indicators, except TSF among men: *P*-value = 0.002). This Figure 1 suggests that adiposity values were higher among participants taking no stairs in daily life, but doing sports ≥1×/week, than among persons taking stairs, but doing sports <1×/week. The same progressive decrease in median adiposity values across categories of PA was observed for all five indicators and in both sexes (Additional file 2). The prevalence of obesity (BMI ≥ 30.0 kg/m^2^) was respectively 45.6%, 41.4%, 19.3%, and 17.6% in men, respectively 44.8%, 41.2%, 21.6%, and 11.0% in women in categories “no stairs, sports < once/week”, “no stairs, sports ≥ once/week”, “stairs, sports < once/week”, and “stairs, sports ≥ once/week” (chi-square and univariate test for trend *P*-values <0.001 in both sexes). Corresponding prevalence estimates for abdominal obesity were 69.7%, 62.1%, 44.0%, and 34.2% in men, respectively 69.0%, 68.6%, 42.3%, and 32.7% in women (chi-square and univariate test for trend *P*-values <0.001 in both sexes).

According to Table 4, “sufficient protein intake” was positively linked with BMI, WC, and WHR in men*.* FV intake was positively related to BMI, SISF, and TSF in women. Except for SISF and TSF in men (with similar *ß* coefficients across PA categories), the gradual decrease in adiposity across categories of increasing PA observed in the Figure 1 was confirmed with multiple adjustment in both sexes (Table 4)*.* Unlike men, women living alone had slightly lower BMI and TSF values than women living with someone. Current smoking was strongly significantly negatively associated with all adiposity indicators but WHR in women. Additional adjustment for number of chronic diseases and self-rated health yielded similar results (Additional file 3).

## Discussion

In this community-dwelling population aged 65–70, overweight and obesity affected more than three quarters of men, and between half and two thirds of women. Abdominal obesity almost hit half of both sexes. Normal weight abdominal obesity, defined as BMI < 25 kg/m^2^ and WC ≥102 cm in men, 88 cm in women, was absent among men, and very rare among women. A gradual decrease in adiposity was almost consistently observed across categories of increasing PA. Adiposity values were higher among participants taking no stairs in daily life, but doing sports ≥1×/week, than among persons taking stairs, but doing sports <1×/week. In both sexes, eating FV ≥2×/day, taking stairs every day, and doing sports ≥ once a week were strongly negatively associated with financial difficulties, and positively with education. The independent and significant negative association of adiposity with PA and education in Lc65+ might be explained by higher fat and total energy intake among less educated persons [33]; these nutritional items were not assessed. A large European cross-sectional study reported a strong association between low levels of PA (during work and leisure time) and obesity, while adjusting for educational level and total energy intake [34].

The main limitations of the present study include the lack of data on total energy intake, fat intake, and sugar intake (the MNA does not include any question on dessert or sugar); the lack of information on total energy intake limited the interpretation of the results, in particular for Table 4. MNA items are categorical and do not allow any quantitative estimation of food intake. Therefore, the present report only presents a few items about eating habits, and does not provide a real dietary assessment. Daily PA and sports frequency were self-reported; a measurement bias could have occurred if participants with higher adiposity values had over-reported their sports frequency, but not their daily PA. To our knowledge, no such a systematic differential bias has previously been described. These analyses could be replicated in studies objectively measuring daily PA and sports frequency. The number of stairs was not specified in the question asked to the participants, nor the number of minutes spent on each of the sports (except that it was at least 20 minutes per session). Therefore, it is not precise enough to clearly establish that there is a dose–response relationship. In addition, the specific benefits of walking and using stairs could not be precisely separated and compared, since a single question addressed both these activities. The sample size was relatively small, thus limiting the generalizability of the findings. This analysis should be replicated in larger samples. The present analysis was cross-sectional: low PA could lead to adiposity [35,36], in the same way as adiposity could lead to low PA [37,38]. The relationship between PA and adiposity should be studied longitudinally. A Swiss study observed that encouraging hospital employees to use stairs instead of elevators during their daily work routine significantly improved cardio-vascular disease risk factors (including WC, body weight, and fat mass) and increased cardiorespiratory fitness after 12 weeks [35]. On the other hand, a small study (involving adults younger than 65 years) reported that experimental weight gain (with overfeeding) reduced objectively measured daily walking distance [37]. In another longitudinal study [38], weight, BMI, fat mass, and WC predicted sedentary time after 5.6 years of follow-up, whereas sedentary time did not predict obesity. With increasing age, visceral abdominal fat mass increases [4,8] while subcutaneous fat mass decreases. Increases in fat mass might not be reflected in proportional increases in anthropometric indicators [4]. However, according to Flegal *et al*. [39], BMI and WC may be inaccurate measures of percentage body fat for an individual, but they correspond well overall with percentage body fat within sex-age groups and distinguish categories of percentage body fat.

Strengths of Lc65+ include the randomly selected sample, which is representative of the general community-dwelling population of Lausanne aged 65 to 70. Its age distribution is homogeneous, reducing the potential for age-related biases (e.g. selection bias) or confounders. This cohort study offers a detailed description of the socio-economic circumstances of this age group, which deserves careful attention for planning health services for the next decade. Height and weight were measured. Moreover, five anthropometric adiposity indicators were assessed, allowing examining the consistency of associations.

Lc65+ obesity prevalence estimates are slightly higher than those observed in the same city in CoLaus study [2], with a prevalence of overweight, obesity, and abdominal obesity of 50%, 23%, and 40% in men aged 65–75 years, respectively 35%, 17% and 45% in women (same definitions in CoLaus and Lc65+). In the Swiss Health Survey (SHS) 2012 [3], the prevalence of overweight and obesity (self-reported height and weight) was 49% and 17% in men, respectively 34% and 14% in women aged 65–74 years at a national level. However, BMI calculated from self-reported height and weight is underestimated [40,41]. Studies allowing reliable international comparisons of overweight and obesity prevalence after age 65 years are lacking because of methodological issues (samples not always representative of the general population, differing participation rates, heterogeneous age distributions, measurements versus self-reports of weight and height) [42]. Despite these limitations, several reports suggest that overweight and obesity prevalence among Swiss adults is lower than in other European countries [40-42]. Several studies have described associations between low socioeconomic status and suboptimal diet [12] and PA [13,43], as well as the links of these behaviors with odds for being overweight or obese [44]. In Lc65+, living alone was associated with eating less FV among men, but less meat among women, a finding already observed in the SHS, and in England [45]. Lc65+ women living alone were also leaner; in a Swedish cohort [46], women “co-habitating” experienced a higher increase in weight and body fat since age 20. Current smoking was consistently negatively associated with adiposity among Lc65+ women, a relationship also observed in CoLaus [2]. As regards daily PA, it has already been observed that pedestrians in lower socioeconomic areas are less likely to climb stairs and more often choose escalators than pedestrians in high socioeconomic areas [43]. Still, a stair climbing intervention was equally effective in both areas [43].

## Conclusions

While the prevalence of abdominal obesity was 45% in both sexes, indicating an important risk of cardio-vascular disease [47], 20% of men and 25% of women avoided using stairs or carrying loads. Whereas obese older persons should be encouraged to practice sports more than once a week [10], the importance of keeping a high level of mobility in daily life should not be overlooked [35]. In this population, eating habits and PA had strong links with socioeconomic factors, which could be the target of public health interventions [43]. Simple measures about use of stairs in everyday life can provide interesting information on health behaviors. In the present study, these measures had consistent cross-sectional associations with adiposity indicators. In conclusion, this study suggests that walking and using stairs in daily life has stronger negative links with adiposity than doing sports at least once a week.

### Ethics approval

The ethics committee of the Faculty of Biology and Medicine of the University of Lausanne has approved the study protocol.

## Abbreviations

BMI: Body mass index; CI: Confidence intervals; FV: Fruits or vegetables; MNA: Mini nutritional assessment; OR: Odds ratio; PA: Physical activity; SHS: Swiss Health Survey; SISF: Supra-iliac skin-fold; TSF: Triceps skin-fold; WC: Waist circumference; WHR: Waist-to-hip ratio.

## Competing interests

The authors declare that they have no competing interests.

## Authors’ contributions

Study design: SE. Data acquisition: SE. Data analysis and interpretation: DH, SE. Manuscript preparation: DH, SE. Both authors read and approved the final manuscript.

## Pre-publication history

The pre-publication history for this paper can be accessed here:

http://www.biomedcentral.com/1471-2458/13/1175/prepub

## Supplementary Material

Additional file 1 Associations between variable “Daily PA and sports” and socio-economic factors and lifestyle factors. Click here for file

Additional file 2 Median values of the 5 adiposity indicators according to variable “Daily PA and sports”. Click here for file

Additional file 3 **Associations of adiposity with diet and physical activity, adjusted for number of chronic diseases.** Associations of adiposity with diet and physical activity, adjusted for self-rated health. Click here for file

## References

[B1] Marques-Vidal P Bovet P Paccaud F Chiolero A Changes of overweight and obesity in the adult Swiss population according to educational level, from 1992 to 2007 BMC Public Health 2010 13 87 10.1186/1471-2458-10-87 20170554 PMC2831837

[B2] Marques-Vidal P Bochud M Mooser V Paccaud F Waeber G Vollenweider P Prevalence of obesity and abdominal obesity in the Lausanne population BMC Public Health 2008 13 330 10.1186/1471-2458-8-330 18816372 PMC2563005

[B3] Body mass index weight categories, Swiss Health Survey 2012 http://www.bfs.admin.ch/bfs/portal/fr/index/themen/14/02/02/key/02.html

[B4] Zamboni M Mazzali G Obesity in the elderly: an emerging health issue Int J Obes (Lond) 2012 13 1151 1152 10.1038/ijo.2012.120 22964828

[B5] Danon-Hersch N Rodondi N Spagnoli J Santos-Eggimann B Prefrailty and chronic morbidity in the youngest old: an insight from the Lausanne cohort Lc65+ J Am Geriatr Soc 2012 13 1687 1694 10.1111/j.1532-5415.2012.04113.x 22906300

[B6] Cruz-Jentoft AJ Baeyens JP Bauer JM Boirie Y Cederholm T Landi F Martin FC Michel JP Rolland Y Schneider SM Sarcopenia: European consensus on definition and diagnosis: Report of the European Working Group on Sarcopenia in Older People Age Ageing 2010 13 412 423 10.1093/ageing/afq034 20392703 PMC2886201

[B7] Stenholm S Alley D Bandinelli S Griswold ME Koskinen S Rantanen T Guralnik JM Ferrucci L The effect of obesity combined with low muscle strength on decline in mobility in older persons: results from the InCHIANTI study Int J Obes (Lond) 2009 13 635 644 10.1038/ijo.2009.62 19381155 PMC2697265

[B8] Atlantis E Martin SA Haren MT Taylor AW Wittert GA Florey Adelaide Male Aging S Lifestyle factors associated with age-related differences in body composition: the Florey Adelaide Male Aging Study Am J Clin Nutr 2008 13 95 104 18614729 10.1093/ajcn/88.1.95

[B9] Zhang X Shu XO Xiang YB Yang G Li H Gao J Cai H Gao YT Zheng W Cruciferous vegetable consumption is associated with a reduced risk of total and cardiovascular disease mortality Am J Clin Nutr 2011 13 240 246 10.3945/ajcn.110.009340 21593509 PMC3127519

[B10] Villareal DT Chode S Parimi N Sinacore DR Hilton T Armamento-Villareal R Napoli N Qualls C Shah K Weight loss, exercise, or both and physical function in obese older adults N Engl J Med 2011 13 1218 1229 10.1056/NEJMoa1008234 21449785 PMC3114602

[B11] Chodzko-Zajko WJ Proctor DN Fiatarone Singh MA Minson CT Nigg CR Salem GJ Skinner JS American College of Sports M American College of Sports Medicine position stand. Exercise and physical activity for older adults Med Sci Sports Exerc 2009 13 1510 1530 10.1249/MSS.0b013e3181a0c95c 19516148

[B12] Watt HC Carson C Lawlor DA Patel R Ebrahim S Influence of life course socioeconomic position on older women’s health behaviors: findings from the British Women’s Heart and Health Study Am J Public Health 2009 13 320 327 10.2105/AJPH.2007.129288 19059863 PMC2622782

[B13] Chad KE Reeder BA Harrison EL Ashworth NL Sheppard SM Schultz SL Bruner BG Fisher KL Lawson JA Profile of physical activity levels in community-dwelling older adults Med Sci Sports Exerc 2005 13 1774 1784 10.1249/01.mss.0000181303.51937.9c 16260980

[B14] de Abreu D Guessous I Vaucher J Preisig M Waeber G Vollenweider P Marques-Vidal P Low compliance with dietary recommendations for food intake among adults Clin Nutr 2013 13 783 8 10.1016/j.clnu.2012.11.022 23260749

[B15] Hendrie GA Coveney J Cox D Exploring nutrition knowledge and the demographic variation in knowledge levels in an Australian community sample Public Health Nutr 2008 13 1365 1371 10.1017/S1368980008003042 18671887

[B16] Beydoun MA Powell LM Wang Y The association of fast food, fruit and vegetable prices with dietary intakes among US adults: is there modification by family income? Soc Sci Med 2008 13 2218 2229 10.1016/j.socscimed.2008.01.018 18313824 PMC4863648

[B17] Poehlman ET Toth MJ Bunyard LB Gardner AW Donaldson KE Colman E Fonong T Ades PA Physiological predictors of increasing total and central adiposity in aging men and women Arch Intern Med 1995 13 2443 2448 10.1001/archinte.1995.00430220101011 7503603

[B18] Walls HL Stevenson CE Mannan HR Abdullah A Reid CM McNeil JJ Peeters A Comparing trends in BMI and waist circumference Obesity (Silver Spring) 2011 13 216 219 10.1038/oby.2010.149 20559295

[B19] Santos-Eggimann B Karmaniola A Seematter-Bagnoud L Spagnoli J Bula C Cornuz J Rodondi N Vollenweider P Waeber G Pecoud A The Lausanne cohort Lc65+: a population-based prospective study of the manifestations, determinants and outcomes of frailty BMC Geriatr 2008 13 20 10.1186/1471-2318-8-20 18706113 PMC2532683

[B20] Chen X Huang Y Cheng HG Lower intake of vegetables and legumes associated with cognitive decline among illiterate elderly Chinese: a 3-year cohort study J Nutr Health Aging 2012 13 549 552 10.1007/s12603-012-0023-2 22659995 PMC12878072

[B21] Folstein MF Folstein SE McHugh PR “Mini-mental state”: a practical method for grading the cognitive state of patients for the clinician J Psychiatr Res 1975 13 189 198 10.1016/0022-3956(75)90026-6 1202204

[B22] Vellas B Villars H Abellan G Soto ME Rolland Y Guigoz Y Morley JE Chumlea W Salva A Rubenstein LZ Garry P Overview of the MNA–Its history and challenges J Nutr Health Aging 2006 13 456 463 17183418

[B23] Wietlisbach V Paccaud F Rickenbach M Gutzwiller F Trends in cardiovascular risk factors (1984–1993) in a Swiss region: results of three population surveys Prev Med 1997 13 523 533 10.1006/pmed.1997.0167 9245675

[B24] Sequeira MM Rickenbach M Wietlisbach V Tullen B Schutz Y Physical activity assessment using a pedometer and its comparison with a questionnaire in a large population survey Am J Epidemiol 1995 13 989 999 7572981 10.1093/oxfordjournals.aje.a117748

[B25] Heymsfield SB Heo M Pietrobelli A Are adult body circumferences associated with height? Relevance to normative ranges and circumferential indexes Am J Clin Nutr 2011 13 302 307 10.3945/ajcn.110.005132 21123461

[B26] Lean ME Han TS Morrison CE Waist circumference as a measure for indicating need for weight management BMJ 1995 13 158 161 10.1136/bmj.311.6998.158 7613427 PMC2550221

[B27] Kaplan GA Haan MN Wallace RB Understanding changing risk factor associations with increasing age in adults Annu Rev Public Health 1999 13 89 108 10.1146/annurev.publhealth.20.1.89 10352851

[B28] Payne ME Steck SE George RR Steffens DC Fruit, vegetable, and antioxidant intakes are lower in older adults with depression J Acad Nutr Diet 2012 13 2022 2027 10.1016/j.jand.2012.08.026 23174689 PMC3520090

[B29] Dvorak RD Del Gaizo AL Engdahl RM Eliason CJ Tobacco use and body mass index: mediated effects through physical inactivity J Health Psychol 2009 13 919 923 10.1177/1359105309341005 19786518

[B30] Arroll B Khin N Kerse N Screening for depression in primary care with two verbally asked questions: cross sectional study BMJ 2003 13 1144 1146 10.1136/bmj.327.7424.1144 14615341 PMC261815

[B31] Ledikwe JH Smiciklas-Wright H Mitchell DC Jensen GL Friedmann JM Still CD Nutritional risk assessment and obesity in rural older adults: a sex difference Am J Clin Nutr 2003 13 551 558 12600842 10.1093/ajcn/77.3.551

[B32] Wu CH Yao WJ Lu FH Yang YC Wu JS Chang CJ Sex differences of body fat distribution and cardiovascular dysmetabolic factors in old age Age Ageing 2001 13 331 336 10.1093/ageing/30.4.331 11509312

[B33] Beydoun MA Wang Y How do socio-economic status, perceived economic barriers and nutritional benefits affect quality of dietary intake among US adults? Eur J Clin Nutr 2008 13 303 313 10.1038/sj.ejcn.1602700 17342164 PMC4887142

[B34] Besson H Ekelund U Luan J May AM Sharp S Travier N Agudo A Slimani N Rinaldi S Jenab M A cross-sectional analysis of physical activity and obesity indicators in European participants of the EPIC-PANACEA study Int J Obes (Lond) 2009 13 497 506 10.1038/ijo.2009.25 19223851

[B35] Meyer P Kayser B Kossovsky MP Sigaud P Carballo D Keller PF Martin XE Farpour-Lambert N Pichard C Mach F Stairs instead of elevators at workplace: cardioprotective effects of a pragmatic intervention Eur J Cardiovasc Prev Rehabil 2010 13 569 575 10.1097/HJR.0b013e328338a4dd 20299999

[B36] Ekelund U Besson H Luan J May AM Sharp SJ Brage S Travier N Agudo A Slimani N Rinaldi S Physical activity and gain in abdominal adiposity and body weight: prospective cohort study in 288,498 men and women Am J Clin Nutr 2011 13 826 835 10.3945/ajcn.110.006593 21346093

[B37] Levine JA McCrady SK Lanningham-Foster LM Kane PH Foster RC Manohar CU The role of free-living daily walking in human weight gain and obesity Diabetes 2008 13 548 554 10.2337/db07-0815 18003759

[B38] Ekelund U Brage S Besson H Sharp S Wareham NJ Time spent being sedentary and weight gain in healthy adults: reverse or bidirectional causality? Am J Clin Nutr 2008 13 612 617 18779275 10.1093/ajcn/88.3.612

[B39] Flegal KM Shepherd JA Looker AC Graubard BI Borrud LG Ogden CL Harris TB Everhart JE Schenker N Comparisons of percentage body fat, body mass index, waist circumference, and waist-stature ratio in adults Am J Clin Nutr 2009 13 500 508 10.3945/ajcn.2008.26847 19116329 PMC2647766

[B40] Peytremann-Bridevaux I Faeh D Santos-Eggimann B Prevalence of overweight and obesity in rural and urban settings of 10 European countries Prev Med 2007 13 442 446 10.1016/j.ypmed.2006.11.011 17258803

[B41] Berghofer A Pischon T Reinhold T Apovian CM Sharma AM Willich SN Obesity prevalence from a European perspective: a systematic review BMC Public Health 2008 13 200 10.1186/1471-2458-8-200 18533989 PMC2441615

[B42] Haftenberger M Lahmann PH Panico S Gonzalez CA Seidell JC Boeing H Giurdanella MC Krogh V Bueno-de-Mesquita HB Peeters PH Overweight, obesity and fat distribution in 50- to 64-year-old participants in the European Prospective Investigation into Cancer and Nutrition (EPIC) Public Health Nutr 2002 13 1147 1162 10.1079/PHN2002396 12639224

[B43] Ryan J Lyon K Webb OJ Eves FF Ryan CG Promoting physical activity in a low socioeconomic area: results from an intervention targeting stair climbing Prev Med 2011 13 352 354 10.1016/j.ypmed.2011.03.004 21397629

[B44] Meyer AM Evenson KR Couper DJ Stevens J Pereria MA Heiss G Television, physical activity, diet, and body weight status: the ARIC cohort Int J Behav Nutr Phys Act 2008 13 68 10.1186/1479-5868-5-68 19091124 PMC2647944

[B45] Donkin AJ Johnson AE Morgan K Neale RJ Page RM Silburn RL Gender and living alone as determinants of fruit and vegetable consumption among the elderly living at home in urban Nottingham Appetite 1998 13 39 51 10.1006/appe.1997.0110 9500802

[B46] Lahmann PH Lissner L Gullberg B Berglund G Sociodemographic factors associated with long-term weight gain, current body fatness and central adiposity in Swedish women Int J Obes Relat Metab Disord 2000 13 685 694 10.1038/sj.ijo.0801219 10878674

[B47] Zhang C Rexrode KM van Dam RM Li TY Hu FB Abdominal obesity and the risk of all-cause, cardiovascular, and cancer mortality: sixteen years of follow-up in US women Circulation 2008 13 1658 1667 10.1161/CIRCULATIONAHA.107.739714 18362231

