# Supplementary material for: Physical activity in daily life is associated with lower adiposity values than doing weekly sports in Lc65+ cohort at baseline
Source: BMC Public Health. 2013 Dec 13;13:1175. doi: 10.1186/1471-2458-13-1175 (PMC3909343; doi:10.1186/1471-2458-13-1175)
Supplement: Additional file 1 — Associations between variable “Daily PA and sports” and socio-economic factors and lifestyle factors. [file 1471-2458-13-1175-S1.docx]

**Additional file 1. Associations between variable “Daily PA and sports” and socio-economic factors**

Univariate associations of the 4 categories of variable “Daily PA and sports” with age, living arrangement, financial difficulties, symptoms of depression, education, and smoking status

|  | **No stairs, no sport** | **No stairs, sports weekly** | **Stairs, no sport** | **Stairs, sports weekly** | **χ^2^ *P*-value** | **Univariate test for trend *P*-** |
| --- | --- | --- | --- | --- | --- | --- |
|  | N(%) | N(%) | N(%) | N(%) |  | **value** |
| **MEN** (Total N=498): N | 69 | 29 | 177 | 223 |  |  |
| Birth year |  |  |  |  | 0.724 | 0.500 |
| 1934 | 12 (17.4 ) | 6 (20.7 ) | 30 (17.0 ) | 38 (17.0 ) |  |  |
| 1935 | 12 (17.4 ) | 8 (27.6 ) | 35 (19.8 ) | 51 (22.9 ) |  |  |
| 1936 | 15 (21.7 ) | 1 (3.5 ) | 38 (21.5 ) | 47 (21.1 ) |  |  |
| 1937 | 13 (18.8 ) | 8 (27.6 ) | 42 (23.7 ) | 43 (19.3 ) |  |  |
| 1938 | 17 (24.6 ) | 6 (20.7 ) | 32 (18.1 ) | 44 (19.7 ) |  |  |
| Living alone | 23 (33.3 ) | 5 (17.2 ) | 32 (18.1 ) | 32 (14.4 ) | 0.005 | 0.001 |
| Financial difficulties (0/1) ‡ | 30 (43.5 ) | 8 (27.6 ) | 47 (26.6 ) | 34 (15.3 ) | <0.001 | <0.001 |
| Symptoms of depression (0/1) | 22 (32.4 ) | 4 (14.3 ) | 31 (17.7 ) | 36 (16.4 ) | 0.023 | 0.011 |
| Education |  |  |  |  | 0.281**†** | 0.010 |
| Basic compulsory | 13 (18.8 ) | 6 (21.4 ) | 29 (16.5 ) | 29 (13.0 ) |  |  |
| Apprenticeship | 36 (52.2 ) | 12 (42.9 ) | 76 (43.2 ) | 92 (41.3 ) |  |  |
| High school or more | 20 (29.0 ) | 10 (35.7 ) | 71 (40.3 ) | 102 (45.7 ) |  |  |
| Current smoking (0/1) § | 23 (33.8) | 6 (21.4) | 49 (27.7) | 34 (15.4) | 0.003 | 0.001 |
| **WOMEN** (Total N=690): N | 116 | 51 | 242 | 281 |  |  |
| Birth year |  |  |  |  | 0.009 | 0.178 |
| 1934 | 22 (19.0 ) | 19 (37.3 ) | 56 (23.1 ) | 41 (14.6 ) |  |  |
| 1935 | 20 (17.2 ) | 8 (15.7 ) | 52 (21.5 ) | 64 (22.8 ) |  |  |
| 1936 | 28 (24.1 ) | 9 (17.7 ) | 54 (22.3 ) | 48 (17.1 ) |  |  |
| 1937 | 20 (17.2 ) | 9 (17.7 ) | 34 (14.1 ) | 65 (23.1 ) |  |  |
| 1938 | 26 (22.4 ) | 6 (11.8 ) | 46 (19.0 ) | 63 (22.4 ) |  |  |
|  |  |  |  |  |  | *Continued…* |
| Living alone | 65 (56.0 ) | 23 (45.1 ) | 109 (45.0 ) | 124 (44.1 ) | 0.163 | 0.046 |
| Financial difficulties (0/1) ‡ | 44 (37.9 ) | 19 (37.3 ) | 74 (30.6 ) | 47 (16.7 ) | <0.001 | <0.001 |
| Symptoms of depression (0/1) | 45 (39.8 ) | 18 (35.3 ) | 57 (23.8 ) | 64 (23.1 ) | 0.002 | <0.001 |
| Education |  |  |  |  | 0.008 | <0.001 |
| Basic compulsory | 44 (37.9 ) | 16 (31.4 ) | 72 (29.9 ) | 62 (22.2 ) |  |  |
| Apprenticeship | 47 (40.5 ) | 18 (35.3 ) | 90 (37.3 ) | 103 (36.9 ) |  |  |
| High school or more | 25 (21.6 ) | 17 (33.3 ) | 79 (32.8 ) | 114 (40.9 ) |  |  |
| Current smoking (0/1) § | 28 (24.4) | 11 (22.0) | 63 (26.1) | 31 (11.0) | <0.001 | 0.001 |

*P<0.05; **P<0.01; ***P<0.001.

**†** Fisher exact two-tailed test (if expected frequency <5 in any of the cells).

‡ Financial difficulties were considered if any of the following criteria was fulfilled: 1) current income clearly lower than others, 2) sometimes difficulty to make ends meet, 3) subsidy for health insurance, or 4) complementary subsidy (from old age insurance).

§ Participants who had stopped smoking before less than one year are considered current smokers in the analyses.
