# Supplementary material for: Physical activity in daily life is associated with lower adiposity values than doing weekly sports in Lc65+ cohort at baseline
Source: BMC Public Health. 2013 Dec 13;13:1175. doi: 10.1186/1471-2458-13-1175 (PMC3909343; doi:10.1186/1471-2458-13-1175)
Supplement: Additional file 2 — Median values of the 5 adiposity indicators according to variable “Daily PA and sports”. [file 1471-2458-13-1175-S2.pdf]

ADDITIONAL FILE 2. MEDIAN VALUES OF THE 5 ADIPOSITY INDICATORS ACCORDING TO VARIABLE "DAILY PA AND SPORTS"

	No stairs, no sport	No stairs, sports weekly	Stairs, no sport	Stairs, sports weekly
	Median	Median	Median	Median
**Men**				
N	66-68	28-29	175-177	222
Body mass index (kg/m^2)	29.1	28.8	27.1	26.7
Waist circumference (cm)	108.0	105.0	100.7	97.8
Waist-to-hip ratio	1.004	0.999	0.971	0.954
Supra-iliac skin-fold (mm)	17.3	13.0	12.6	12.4
Triceps skin-fold (mm)	12.3	10.8	10.6	10.3
**Women**				
N	110-116	50-51	237-241	278-281
Body mass index (kg/m^2)	29.5	27.8	25.6	24.6
Waist circumference (cm)	95.2	93.8	85.0	82.5
Waist-to-hip ratio	0.860	0.863	0.829	0.825
Supra-iliac skin-fold (mm)	19.4	15.7	15.2	13.0
Triceps skin-fold (mm)	23.6	20.4	20.2	19.8

Definition of variable "Daily PA and sports":

No stairs: sitting or lying most of the time, or often walking, but avoiding stairs and loads (versus often walking and using stairs, carrying light loads, and important physical activity, carries heavy loads)

No sport: sports (≥20 minutes) frequency <1x/week (versus ≥1x/week)
