# Supplementary material for: Physical activity in daily life is associated with lower adiposity values than doing weekly sports in Lc65+ cohort at baseline
Source: BMC Public Health. 2013 Dec 13;13:1175. doi: 10.1186/1471-2458-13-1175 (PMC3909343; doi:10.1186/1471-2458-13-1175)
Supplement: Additional file 3 — Associations of adiposity with diet and physical activity, adjusted for number of chronic diseases. Associations of adiposity with diet and physical activity, adjusted for self-rated health. [file 1471-2458-13-1175-S3.docx]

**ADDITIONAL FILE 3. TABLE A. ASSOCIATIONS OF ADIPOSITY WITH DIET AND PHYSICAL ACTIVITY, ADJUSTED FOR NUMBER OF CHRONIC DISEASES.**

The five adiposity indicators have been log-transformed. Multivariate linear regression analysis. The table shows all covariates included in the adjustment.

|  | **Ln(BMI)** | |  | **Ln(WC)** | |  | **Ln(WHR)** |  | | **Ln(SISF)** |  | **Ln(TSF)** |  |
| --- | --- | --- | --- | --- | --- | --- | --- | --- | --- | --- | --- | --- | --- |
|  | ***ß* [95%CI]** | | ***P*** | ***ß* [95%CI]** | | ***P*** | ***ß* [95%CI]** | ***P*** | | ***ß* [95%CI]** | ***P*** | ***ß* [95%CI]** | ***P*** |
| **MEN:(N≥477):** |  | |  |  | |  |  |  | |  |  |  |  |
| Eating habits: |  | |  |  | |  |  |  | |  |  |  |  |
| Three meals/day | -0.01[-0.04-0.01] | |  | 0.00[-0.02-0.02] | |  | -0.01[-0.02-0.00] |  | | 0.01[-0.07-0.08] |  | -0.04[-0.11-0.04] |  |
| Fruit and veg. ≥twice/day | 0.00[-0.03-0.03] | |  | 0.00[-0.02-0.03] | |  | 0.00[-0.02-0.01] |  | | 0.05[-0.05-0.14] |  | 0.03[-0.07-0.12] |  |
| Sufficient protein intake$ | 0.03[0.00-0.06] | | * | 0.03[0.01-0.05] | | ** | 0.02[0.01-0.03] | ** | | 0.04[-0.04-0.12] |  | -0.03[-0.11-0.05] |  |
| Daily PA and sports |  | |  |  | |  |  |  | |  |  |  |  |
| 1: No stairs, no sport | 0 (ref.) | |  | 0 (ref.) | |  | 0 (ref.) |  | | 0 (ref.) |  | 0 (ref.) |  |
| 2: No stairs, sports ≥1x/wk | -0.03[-0.08-0.03] | |  | -0.03[-0.07-0.02] | |  | 0.00[-0.03-0.03] |  | | -0.25[-0.43--0.06] | ** | -0.22[-0.39--0.04] | * |
| 3: Stairs, no sport | -0.07[-0.11--0.03] | | *** | -0.06[-0.09--0.03] | | *** | -0.02[-0.04-0.00] | * | | -0.24[-0.35--0.12] | *** | -0.17[-0.29--0.06] | ** |
| 4: Stairs, sports ≥1x/wk | -0.09[-0.13--0.05] | | *** | -0.09[-0.12--0.06] | | *** | -0.05[-0.07--0.03] | *** | | -0.29[-0.41--0.18] | *** | -0.20[-0.31--0.08] | ** |
| Age (per 1-birth year) | 0.01[0.00-0.01] | |  | 0.00[0.00-0.01] | |  | 0.00[0.00-0.01] |  | | 0.02[0.00-0.05] |  | 0.00[-0.02-0.03] |  |
| Living alone (0/1) | -0.03[-0.06-0.00] | |  | -0.01[-0.04-0.01] | |  | 0.00[-0.01-0.02] |  | | 0.03[-0.07-0.12] |  | 0.02[-0.08-0.11] |  |
| Financial diff. (0/1)€ | -0.01[-0.04-0.02] | |  | 0.00[-0.02-0.02] | |  | 0.00[-0.02-0.01] |  | | -0.01[-0.10-0.08] |  | 0.01[-0.08-0.09] |  |
| Symptoms of depression | 0.03[0.00-0.06] | |  | 0.01[-0.01-0.04] | |  | 0.00[-0.01-0.02] |  | | 0.05[-0.04-0.15] |  | 0.03[-0.06-0.12] |  |
| Education |  | |  |  | |  |  |  | |  |  |  |  |
| Basic compulsory | 0 (ref.) | |  | 0 (ref.) | |  | 0 (ref.) |  | | 0 (ref.) |  | 0 (ref.) |  |
| Apprenticeship | -0.03[-0.07-0.00] | |  | -0.02[-0.05-0.01] | |  | -0.01[-0.03-0.01] |  | | -0.01[-0.11-0.10] |  | -0.03[-0.14-0.07] |  |
| ≥High school | -0.05[-0.09--0.02] | | ** | -0.03[-0.06--0.01] | | * | -0.02[-0.04--0.01] | * | | -0.02[-0.13-0.08] |  | -0.03[-0.14-0.08] |  |
|  |  | |  |  | |  |  |  | |  |  | *Continued…* |  |
| Current smoking (0/1)§ | -0.04[-0.06--0.01] | | * | -0.01[-0.03-0.01] | |  | 0.01[-0.01-0.02] |  | | -0.11[-0.20--0.02] | * | -0.05[-0.14-0.03] |  |
| Number of chronic diseases # |  | |  |  | |  |  |  | |  |  |  |  |
| 0 | 0 (ref.) | |  | 0 (ref.) | |  | 0 (ref.) |  | | 0 (ref.) |  | 0 (ref.) |  |
| 1 | 0.03[0.00-0.05] | |  | 0.02[0.00-0.04] | | * | 0.01[-0.01-0.02] |  | | -0.01[-0.09-0.07] |  | 0.05[-0.03-0.13] |  |
| ≥2 | 0.02[-0.01-0.05] | |  | 0.02[0.00-0.05] | |  | 0.01[0.00-0.03] |  | | 0.02[-0.08-0.11] |  | 0.03[-0.07-0.12] |  |
| **WOMEN (N≥ 657)** |  |  | |  |  | |  | |  |  |  |  |  |
| Eating habits: |  |  | |  |  | |  | |  |  |  |  |  |
| Three meals/day | 0.00[-0.03-0.03] |  | | 0.01[-0.02-0.03] |  | | 0.00[-0.01-0.01] | |  | 0.06[-0.02-0.13] |  | -0.03[-0.09-0.03] |  |
| Fruit and veg. ≥twice/day | 0.05[0.00-0.10] | * | | 0.02[-0.02-0.05] |  | | -0.01[-0.03-0.01] | |  | 0.14[0.02-0.27] | * | 0.14[0.04-0.23] | ** |
| Sufficient protein intake$ | 0.03[0.00-0.06] | * | | 0.02[0.00-0.04] |  | | 0.01[-0.01-0.02] | |  | 0.04[-0.04-0.11] |  | 0.05[-0.01-0.11] |  |
| Daily PA and sports |  |  | |  |  | |  | |  |  |  |  |  |
| 1: No stairs, no sport | 0 (ref.) |  | | 0 (ref.) |  | | 0 (ref.) | |  | 0 (ref.) |  | 0 (ref.) |  |
| 2: No stairs, sports ≥1x/wk | -0.04[-0.10-0.01] |  | | -0.02[-0.07-0.02] |  | | 0.00[-0.03-0.03] | |  | -0.16[-0.32-0.00] | * | -0.13[-0.25--0.01] | * |
| 3: Stairs, no sport | -0.12[-0.16--0.08] | *** | | -0.08[-0.11--0.05] | *** | | -0.02[-0.04--0.01] | | ** | -0.25[-0.36--0.14] | *** | -0.15[-0.23--0.07] | *** |
| 4: Stairs, sports ≥1x/wk | -0.16[-0.20--0.12] | *** | | -0.12[-0.15--0.09] | *** | | -0.03[-0.05--0.01] | | ** | -0.34[-0.45--0.23] | *** | -0.19[-0.27--0.11] | *** |
| Age (per 1-birth year) | 0.00[-0.01-0.01] |  | | 0.00[-0.01-0.01] |  | | 0.00[0.00-0.00] | |  | 0.01[-0.02-0.03] |  | 0.00[-0.02-0.02] |  |
| Living alone (0/1) | -0.03[-0.06-0.00] | * | | -0.02[-0.04-0.00] |  | | -0.01[-0.02-0.01] | |  | -0.05[-0.12-0.03] |  | -0.06[-0.11-0.00] | * |
| Financial diff. (0/1)€ | 0.04[0.01-0.07] | * | | 0.02[-0.01-0.04] |  | | 0.00[-0.01-0.02] | |  | 0.08[0.00-0.16] |  | 0.05[-0.01-0.12] |  |
| Symptoms of depression | 0.01[-0.02-0.04] |  | | 0.01[-0.01-0.04] |  | | 0.01[-0.01-0.02] | |  | 0.06[-0.02-0.14] |  | 0.01[-0.05-0.07] |  |
| Education |  |  | |  |  | |  | |  |  |  |  |  |
| Basic compulsory | 0 (ref.) |  | | 0 (ref.) |  | | 0 (ref.) | |  | 0 (ref.) |  | 0 (ref.) |  |
| Apprenticeship | -0.02[-0.06-0.01] |  | | -0.01[-0.04-0.02] |  | | -0.01[-0.02-0.01] | |  | -0.04[-0.13-0.04] |  | -0.02[-0.09-0.05] |  |
| ≥High school | -0.06[-0.10--0.03] | *** | | -0.04[-0.07--0.01] | ** | | -0.02[-0.03-0.00] | | * | -0.14[-0.23--0.05] | ** | -0.07[-0.14-0.00] |  |
|  |  |  | |  |  | |  | |  |  |  | *Continued…* |  |
| Current smoking (0/1)§ | -0.07[-0.11--0.04] | *** | | -0.04[-0.06--0.01] | ** | | 0.00[-0.01-0.02] | |  | -0.21[-0.30--0.12] | *** | -0.15[-0.22--0.08] | *** |
| Number of chronic diseases # |  |  | |  |  | |  | |  |  |  |  |  |
| 0 | 0 (ref.) |  | | 0 (ref.) |  | | 0 (ref.) | |  | 0 (ref.) |  | 0 (ref.) |  |
| 1 | 0.02[-0.01-0.06] |  | | 0.02[-0.01-0.04] |  | | 0.01[-0.01-0.02] | |  | 0.04[-0.04-0.13] |  | 0.03[-0.03-0.10] |  |
| ≥2 | 0.01[-0.03-0.04] |  | | 0.02[-0.01-0.05] |  | | 0.01[0.00-0.03] | |  | 0.00[-0.09-0.09] |  | 0.02[-0.05-0.09] |  |

$ “Sufficient protein intake” was defined if the participant reported eating meat, fish or poultry every day, or as an alternative, if he consumed dairy products ≥ once a day and eggs or leguminous plants ≥twice/week.

€ Financial difficulties (diff.) are considered if any of the following criteria is fulfilled: 1) current income clearly lower than others, 2) sometimes difficulty to make ends meet, 3) subsidy for health insurance, or 4) complementary subsidy (from old age insurance).

§ Participants who had stopped smoking before less than one year are considered current smokers in the analyses.

*P<0.05; **P<0.01; ***P<0.001.

# All chronic diseases are self-reported medical diagnoses (“Has a doctor ever told you that you had…?”). The number of chronic diseases included coronary

heart disease, other heart diseases (congestive heart failure, cardiac valvular disease, and heart muscle disease), stroke, diabetes mellitus, chronic respiratory

disease, osteoporosis, arthritis, cancer, and depression (maximum number = 9).

**TABLE B. ASSOCIATIONS OF ADIPOSITY WITH DIET AND PHYSICAL ACTIVITY, ADJUSTED FOR SELF-RATED HEALTH**

The five adiposity indicators have been log-transformed. Multivariate linear regression analysis. The table shows all covariates included in the adjustment.

|  | **Ln(BMI)** | |  | **Ln(WC)** | |  | **Ln(WHR)** |  | | **Ln(SISF)** |  | **Ln(TSF)** |  |
| --- | --- | --- | --- | --- | --- | --- | --- | --- | --- | --- | --- | --- | --- |
|  | ***ß* [95%CI]** | | ***P*** | ***ß* [95%CI]** | | ***P*** | ***ß* [95%CI]** | ***P*** | | ***ß* [95%CI]** | ***P*** | ***ß* [95%CI]** | ***P*** |
| **MEN:(N≥476):** |  | |  |  | |  |  |  | |  |  |  |  |
| Eating habits: |  | |  |  | |  |  |  | |  |  |  |  |
| Three meals/day | -0.02[-0.04-0.01] | |  | 0.00[-0.02-0.01] | |  | -0.01[-0.02-0.00] |  | | 0.00[-0.07-0.08] |  | -0.04[-0.11-0.03] |  |
| Fruit and veg. ≥twice/day | 0.01[-0.03-0.04] | |  | 0.01[-0.02-0.03] | |  | 0.00[-0.02-0.01] |  | | 0.06[-0.04-0.15] |  | 0.04[-0.06-0.13] |  |
| Sufficient protein intake$ | 0.03[0.00-0.06] | | * | 0.04[0.02-0.06] | | ** | 0.02[0.01-0.03] | ** | | 0.05[-0.03-0.13] |  | -0.02[-0.10-0.06] |  |
| Daily PA and sports |  | |  |  | |  |  |  | |  |  |  |  |
| 1: No stairs, no sport | 0 (ref.) | |  | 0 (ref.) | |  | 0 (ref.) |  | | 0 (ref.) |  | 0 (ref.) |  |
| 2: No stairs, sports ≥1x/wk | -0.02[-0.08-0.04] | |  | -0.03[-0.07-0.02] | |  | 0.00[-0.03-0.03] |  | | -0.23[-0.41--0.05] | * | -0.20[-0.37--0.02] | * |
| 3: Stairs, no sport | -0.07[-0.11--0.03] | | *** | -0.05[-0.08--0.03] | | *** | -0.02[-0.04-0.00] | * | | -0.23[-0.35--0.11] | *** | -0.15[-0.27--0.04] | * |
| 4: Stairs, sports ≥1x/wk | -0.09[-0.12--0.05] | | *** | -0.08[-0.11--0.05] | | *** | -0.05[-0.07--0.03] | *** | | -0.28[-0.40--0.16] | *** | -0.17[-0.29--0.05] | ** |
| Age (per 1-birth year) | 0.01[0.00-0.02] | |  | 0.00[0.00-0.01] | |  | 0.00[0.00-0.01] |  | | 0.02[0.00-0.05] |  | 0.00[-0.03-0.02] |  |
| Living alone (0/1) | -0.03[-0.06-0.00] | |  | -0.01[-0.04-0.01] | |  | 0.01[-0.01-0.02] |  | | 0.03[-0.07-0.12] |  | 0.02[-0.08-0.11] |  |
| Financial diff. (0/1)€ | -0.01[-0.04-0.02] | |  | 0.00[-0.02-0.02] | |  | 0.00[-0.01-0.02] |  | | -0.01[-0.09-0.08] |  | 0.01[-0.08-0.09] |  |
| Symptoms of depression | 0.03[0.00-0.06] | |  | 0.01[-0.01-0.03] | |  | 0.00[-0.01-0.02] |  | | 0.05[-0.04-0.15] |  | 0.02[-0.07-0.11] |  |
| Education |  | |  |  | |  |  |  | |  |  |  |  |
| Basic compulsory | 0 (ref.) | |  | 0 (ref.) | |  | 0 (ref.) |  | | 0 (ref.) |  | 0 (ref.) |  |
| Apprenticeship | -0.03[-0.06-0.01] | |  | -0.02[-0.04-0.01] | |  | -0.01[-0.03-0.01] |  | | 0.00[-0.10-0.11] |  | -0.02[-0.12-0.09] |  |
| ≥High school | -0.05[-0.08--0.01] | | ** | -0.03[-0.06-0.00] | | * | -0.02[-0.04-0.00] | * | | -0.02[-0.12-0.09] |  | -0.03[-0.13-0.08] |  |
| Current smoking (0/1)§ | -0.04[-0.06--0.01] | | * | -0.01[-0.03-0.01] | |  | 0.00[-0.01-0.02] |  | | -0.11[-0.20--0.02] | * | -0.06[-0.14-0.03] |  |
|  |  | |  |  | |  |  |  | |  |  | *Continued…* |  |
| Self-rated health # |  | |  |  | |  |  |  | |  |  |  |  |
| Clearly better | 0 (ref.) | |  | 0 (ref.) | |  | 0 (ref.) |  | | 0 (ref.) |  | 0 (ref.) |  |
| Slightly better | -0.01[-0.06-0.03] | |  | -0.01[-0.04-0.02] | |  | -0.01[-0.03-0.02] |  | | 0.03[-0.10-0.16] |  | -0.08[-0.21-0.05] |  |
| In the mean | -0.01[-0.05-0.04] | |  | 0.01[-0.02-0.04] | |  | 0.01[-0.01-0.03] |  | | 0.05[-0.08-0.19] |  | 0.00[-0.13-0.14] |  |
| Slightly poorer | -0.01[-0.08-0.06] | |  | 0.03[-0.03-0.08] | |  | 0.00[-0.04-0.04] |  | | -0.07[-0.29-0.15] |  | -0.07[-0.28-0.14] |  |
| Clearly poorer | 0.16[0.00-0.31] | | * | 0.13[0.02-0.25] | | * | 0.00[-0.08-0.07] |  | | 0.63[0.16-1.09] | ** | 0.38[-0.03-0.79] |  |
| **WOMEN (N≥ 657)** |  |  | |  |  | |  | |  |  |  |  |  |
| Eating habits: |  |  | |  |  | |  | |  |  |  |  |  |
| Three meals/day | 0.00[-0.03-0.03] |  | | 0.00[-0.02-0.03] |  | | 0.00[-0.01-0.01] | |  | 0.05[-0.02-0.13] |  | -0.03[-0.09-0.02] |  |
| Fruit and veg. ≥twice/day | 0.05[0.00-0.10] | * | | 0.02[-0.02-0.06] |  | | -0.01[-0.03-0.02] | |  | 0.14[0.01-0.27] | * | 0.13[0.03-0.23] | ** |
| Sufficient protein intake$ | 0.03[0.00-0.06] |  | | 0.02[0.00-0.04] |  | | 0.01[-0.01-0.02] | |  | 0.03[-0.04-0.11] |  | 0.05[-0.01-0.11] |  |
| Daily PA and sports |  |  | |  |  | |  | |  |  |  |  |  |
| 1: No stairs, no sport | 0 (ref.) |  | | 0 (ref.) |  | | 0 (ref.) | |  | 0 (ref.) |  | 0 (ref.) |  |
| 2: No stairs, sports ≥1x/wk | -0.05[-0.11-0.01] |  | | -0.02[-0.07-0.02] |  | | 0.00[-0.03-0.03] | |  | -0.17[-0.33--0.01] | * | -0.14[-0.26--0.02] | * |
| 3: Stairs, no sport | -0.12[-0.16--0.08] | *** | | -0.08[-0.11--0.05] | *** | | -0.02[-0.04--0.01] | | * | -0.26[-0.37--0.15] | *** | -0.16[-0.25--0.08] | *** |
| 4: Stairs, sports ≥1x/wk | -0.16[-0.20--0.12] | *** | | -0.11[-0.15--0.08] | *** | | -0.03[-0.05--0.01] | | ** | -0.34[-0.45--0.23] | *** | -0.20[-0.29--0.12] | *** |
| Age (per 1-birth year) | 0.00[-0.01-0.01] |  | | 0.00[-0.01-0.01] |  | | 0.00[0.00-0.00] | |  | 0.01[-0.02-0.03] |  | 0.00[-0.02-0.02] |  |
| Living alone (0/1) | -0.03[-0.06-0.00] | * | | -0.02[-0.04-0.00] |  | | -0.01[-0.02-0.01] | |  | -0.05[-0.12-0.03] |  | -0.06[-0.11-0.00] | * |
| Financial diff. (0/1)€ | 0.04[0.01-0.07] | * | | 0.02[0.00-0.05] |  | | 0.00[-0.01-0.02] | |  | 0.08[0.00-0.17] |  | 0.06[-0.01-0.12] |  |
| Symptoms of depression | 0.01[-0.02-0.04] |  | | 0.01[-0.01-0.03] |  | | 0.01[-0.01-0.02] | |  | 0.06[-0.02-0.14] |  | 0.02[-0.04-0.08] |  |
| Education |  |  | |  |  | |  | |  |  |  |  |  |
| Basic compulsory | 0 (ref.) |  | | 0 (ref.) |  | | 0 (ref.) | |  | 0 (ref.) |  | 0 (ref.) |  |
| Apprenticeship | -0.02[-0.05-0.01] |  | | -0.01[-0.03-0.02] |  | | -0.01[-0.02-0.01] | |  | -0.04[-0.13-0.05] |  | -0.02[-0.09-0.05] |  |
| ≥High school | -0.06[-0.10--0.03] | *** | | -0.04[-0.06--0.01] | ** | | -0.02[-0.03-0.00] | | * | -0.14[-0.23--0.05] | ** | -0.07[-0.14-0.00] |  |
|  |  |  | |  |  | |  | |  |  |  | *Continued…* |  |
| Current smoking (0/1)§ | -0.07[-0.11--0.04] | *** | | -0.04[-0.06--0.01] | ** | | 0.01[-0.01-0.02] | |  | -0.21[-0.30--0.12] | *** | -0.15[-0.22--0.08] | *** |
| Self-rated health # |  |  | |  |  | |  | |  |  |  |  |  |
| Clearly better | 0 (ref.) |  | | 0 (ref.) |  | | 0 (ref.) | |  | 0 (ref.) |  | 0 (ref.) |  |
| Slightly better | 0.01[-0.04-0.06] |  | | 0.01[-0.03-0.05] |  | | 0.01[-0.02-0.03] | |  | -0.01[-0.15-0.12] |  | 0.00[-0.10-0.11] |  |
| In the mean | 0.02[-0.03-0.07] |  | | 0.03[-0.01-0.07] |  | | 0.02[0.00-0.04] | |  | 0.05[-0.09-0.18] |  | 0.02[-0.08-0.12] |  |
| Slightly poorer | -0.03[-0.10-0.05] |  | | 0.02[-0.04-0.08] |  | | 0.02[-0.01-0.06] | |  | -0.12[-0.32-0.09] |  | -0.15[-0.30-0.01] |  |
| Clearly poorer | 0.15[-0.01-0.31] |  | | 0.11[-0.01-0.24] |  | | 0.02[-0.05-0.10] | |  | 0.19[-0.23-0.61] |  | 0.16[-0.17-0.48] |  |

$ “Sufficient protein intake” was defined if the participant reported eating meat, fish or poultry every day, or as an alternative, if he consumed dairy products ≥ once a day and eggs or leguminous plants ≥twice/week.

€ Financial difficulties (diff.) are considered if any of the following criteria is fulfilled: 1) current income clearly lower than others, 2) sometimes difficulty to make ends meet, 3) subsidy for health insurance, or 4) complementary subsidy (from old age insurance).

§ Participants who had stopped smoking before less than one year are considered current smokers in the analyses.

*P<0.05; **P<0.01; ***P<0.001.

# Self-rated health: *If you compare your current health with persons of your age, do you think that you are …? 1) clearly in better health, 2) slightly in better*

*health, 3) in the mean, 4) slightly in poorer health, 5) clearly in poorer health.*
